# Gene Expression Profiles Modulated by Lipophilic Sea Buckthorn ( *Hippophae rhamnoides* L.) Extract in BT‐549 Triple‐Negative Breast Cancer Cells

**DOI:** 10.1002/fsn3.71112

**Published:** 2025-12-16

**Authors:** Simona Visan, Loredana Balacescu, Flaviu Drigla, Ovidiu Balacescu, Adela Pintea

**Affiliations:** ^1^ Department of Genetics, Genomics, and Experimental Pathology The Oncology Institute “Professor Dr. Ion Chiricuta” Cluj‐Napoca Romania; ^2^ Babes‐Bolyai University Cluj‐Napoca Romania; ^3^ Department of Chemistry and Biochemistry University of Agricultural Sciences and Veterinary Medicine Cluj‐Napoca Romania

**Keywords:** carotenoids, cholesterol biosynthesis, ferroptosis, oxidative stress, sea buckthorn, triple‐negative breast cancer

## Abstract

Triple‐negative breast cancer (TNBC) is an aggressive subtype with high heterogeneity, metastasis, and drug resistance, underscoring the need for new therapeutic strategies. Sea buckthorn berries, long used in traditional medicine, are rich in carotenoids with reported anticancer activity. To investigate their transcriptional effects, we treated BT‐549 TNBC cells with a saponified lipophilic sea buckthorn berry extract (LSBE) and performed gene expression profiling using microarray (four independent replicates of treated, respectively, untreated cells). LSBE altered the expression of 330 protein‐coding genes (111 downregulated, 219 upregulated; fold ratio ±1.5, adj. *p* < 0.05). ingenuity pathway analysis (IPA) revealed activation of cholesterol biosynthesis (*z* = 3.46, *p* = 2.7E−15) and ferroptosis (*z* = 0.78, *p* = 5.3E−09), while oxidative stress (*z* = −2.18, *p* = 3.4E−05) and cholesterol accumulation (*z* = −2.14, *p* = 3.4E−06) were inhibited. Gene set enrichment analysis (GSEA) further indicated upregulation of cholesterol homeostasis and mTORC1 signaling. Our findings suggest that carotenoid‐enriched LSBE modulates key pathways related to cellular homeostasis, antioxidant defense, and apoptosis in TNBC. While limited to transcriptional profiling, this exploratory study provides a foundation for future functional validation of LSBE as a complementary TNBC therapeutic approach.

## Graphical abstract

Transcriptomic profiling showed that carotenoid‐rich lipophilic sea buckthorn extract (LSBE) significantly altered genes controlling cholesterol metabolism, mTORC1 signaling, oxidative stress, and ferroptosis in BT‐549 TNBC cells. These findings generate mechanistic hypotheses for LSBE as a potential complementary approach in TNBC therapy, pending functional validation.

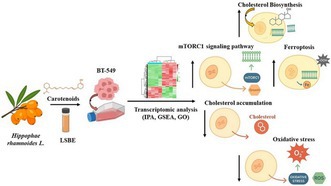

## 1 Introduction

Breast cancer (BC) is the most commonly diagnosed cancer among women and one of the leading causes of cancer‐related mortality in women (Sung et al. 2021). Triple‐negative breast cancer (TNBC) represents a molecular subtype of BC (15%–20%) (Garrido‐Castro et al. 2019) that does not benefit from targeted therapies (anti‐HER2 and anti‐ER), like the other BC molecular subtypes, luminal A/B, or HER2+. Consequently, the standard care for TNBC includes chemotherapy based on platinum compounds or taxanes. However, less than 30% of TNBC patients achieve a complete response (Li et al. 2022), while the survival rate is between 17 and 24 months (Shukla et al. 2019). Considering the increased heterogeneity, high metastasis capacity, and drug resistance of TNBC, it is essential to improve the TNBC treatment by identifying new molecular features to develop targeted therapies or the development of new antitumor compounds.

Sea buckthorn ( *Hippophae rhamnoides* L.) berries have proven to possess many pharmacological and nutritional values due to their abundance of bioactive compounds. They have been used in traditional medicine for centuries, especially by Mongolians and Tibetans (Chandra 2018). The abundance of bioactive compounds is composed of carbohydrates, proteins, flavonoids, antioxidants, sugars, sugar alcohols, fat‐ or water‐soluble vitamins, phytosterols, tocopherols, polyphenols, carotenoids, polyunsaturated fatty acids, amino acids, phenolic acids, tannins, flavonols, and minerals (Wang et al. 2022). Many pharmacological studies have focused on investigating the sea buckthorn berries' health properties, including anticancer (Wu et al. 2021), antioxidant (Xu et al. 2024), anti‐inflammatory (Dudau et al. 2021), and many others. Recent research demonstrated that total circulating carotenoids, mainly α‐carotene, β‐carotene, β‐cryptoxanthin, lycopene, and lutein, were associated with a decreased risk of breast cancer (Dehnavi et al. 2024).

Recent research has shown that dietary supplementation with sea buckthorn reduced triglycerides, total cholesterol, and low‐density lipoprotein cholesterol, only in people with abnormal lipid metabolism, while no adverse effects were recorded on blood sugar, blood pressure, and BMI (Geng et al. 2022). In cancer cells, cholesterol uptake and synthesis rates are usually increased, leading to abnormal metabolism (Cai et al. 2019), while the efflux and esterification of cholesterol affect the formation of tumor cells (Gu et al. 2019). Thus, dyshomeostasis of cholesterol levels has been established as one of the hallmarks of cancer (Hanahan 2022). Furthermore, many studies have shown that ferroptosis, autophagy, tumor cell, and immune cell stemness, and the cellular DNA damage response are regulated through cholesterol metabolism (Xiao et al. 2023). It is essential to maintain homeostasis between cholesterol de novo biosynthesis, uptake, export, and storage (Luo et al. 2020). The results of the research studies that have been carried out to investigate the association between breast cancer risk and circulating cholesterol levels remain debatable due to the positive correlations (Kim et al. 2017) or negative correlations (Poirot 2022), while some studies found no correlations (His et al. 2017). Given the broad spectrum of medicinal properties attributed to sea buckthorn (Ma et al. 2022), and the scarcity of studies addressing the molecular mechanisms modulated by its lipophilic bioactive compounds in TNBC, our exploratory study seeks to fill this important gap. To our knowledge, this is the first transcriptomic investigation of LSBE in TNBC, aimed at elucidating the key signaling pathways, biological processes, cellular functions, and upstream regulators affected in the BT‐549 cell line—a well‐characterized model of an aggressive breast cancer subtype, the mesenchymal stem‐like TNBC associated with chemoresistance and poor clinical outcomes.

## 2 Materials and Methods

### 2.1 Carotenoid Composition of LSBE

The lipophilic saponified sea buckthorn extract (LSBE) was obtained by solvent extraction from sea buckthorn berry pulp, followed by saponification with KOH, as previously described (Visan et al. 2023). Saponification was performed to remove the lipid fraction (mainly triacylglycerols) and to release xanthophylls (zeaxanthin, lutein, and β‐cryptoxanthin) from their esterified forms present in the crude extract. The carotenoid composition of LSBE was determined using C30‐HPLC‐PDA. Carotenoids were identified by comparing retention times with commercial standards, as well as by analyzing UV–VIS spectra and elution orders reported in the literature. Quantification was performed by external calibration with β‐carotene, lutein, β‐cryptoxanthin, and zeaxanthin standards purchased from Extrasynthese (Lyon, France) in the range of 1–100 μg/mL. The major compounds in the LSBE were zeaxanthin (42.6%), all‐trans‐β‐carotene (20.5%), lutein (8.93%), γ‐carotene (8.15%), and β‐cryptoxanthin (4.6%).

### 2.2 Cell Culturing

The human TNBC cell line BT‐549 (ductal carcinoma, mesenchymal subtype, ER‐, PR‐, HER2‐) from American Type Culture Collection (ATCC) was cultured in RPMI‐1640 medium supplemented with 10% FBS, 1% penicillin–streptomycin, 1% glutamine, 0.02% insulin, and maintained at 37°C in a 5% CO_2_ incubator. All cell culture reagents were purchased from Sigma‐Aldrich (St. Louis, MO, USA). In our previous study (Visan et al. 2023), LSBE showed greater cytotoxicity in BT‐549 cells (IC₅₀ = 12.62 μM) compared with T47D cells (IC₅₀ = 19.45 μM), selectively reducing ROS levels under oxidative stress conditions and inducing late‐stage apoptosis (40.6%, *p* = 0.0137). Based on this higher sensitivity and biological relevance, BT‐549 was chosen as the model for the present transcriptomic study.

### 2.3 Treatment

3.5 × 10^5^ cells were seeded in 6‐well plates (TPP, Switzerland) at a density of 10^5^ cells/mL 24 h before treatment. After 24 h, the culture medium was removed and replaced with 3 mL of fresh medium containing 12.62 μM (IC_50_) of LSBE, respectively, fresh culture medium. LSBE was characterized and purified following the protocol described in our previous study. The cells were maintained in these conditions for 24 h and processed for subsequent experiments. For this experiment, four biological replicates were used for treated and untreated cells. All experiments in this study were conducted using the same well‐characterized batch of sea buckthorn extract described in our previous study.

### 2.4 RNA Isolation and Purification

After 24 h of treatment, cells were collected in 800 μL TRI Reagent Solution (Ambion, Thermo Fisher Scientific) and processed for total RNA extraction using the classical phenol‐chloroform method (Sakyi et al. 2023). The isolated RNAs were evaluated quantitatively with the NanoDrop ND‐1000 spectrophotometer (Thermo Scientific, Wilmington, DE, USA) and qualitatively with the 2100 Bioanalyzer (Agilent Technologies, Santa Clara, CA, USA) before further processing. All analyzed samples had RNAs with an RNA integrity number (RIN) between 9 and 10.

### 2.5 Microarray Expression Profiling

200 ng of total RNA was used from each sample to synthesize the microarray probes (cRNA‐Cy3) in two reaction steps using a one‐color Agilent Low Input Quick Amp Labeling Kit according to the manufacturer's recommendations. All labeled cRNAs (Cy3) were purified using an RNeasy Mini kit (Qiagen, Hilden, Germany) and were evaluated for quality control using a Nanodrop ND‐1000 spectrophotometer (Thermo Fischer Scientific, Wilmington, DE, USA). The probes were hybridized for 17 h at 65°C on whole‐human‐genome 4 × 44 K microarray slides (design 014850) according to the manufacturer's protocol (Agilent Technologies, Santa Clara, CA, USA). The slides were scanned with an Agilent G2505C Microarray Scanner (Agilent Technologies, Santa Clara, CA, USA) at 5 μm resolution, and the microarray images were processed with Agilent Feature Extraction (FE) software v. 11.5.1.1 (Agilent Technologies, Santa Clara, CA, USA). Raw median signals from FE were preprocessed and analyzed in R/Bioconductor. After filtering out the control and flagged spots, the data were quantile normalized between arrays, and a median signal value for the replicated transcripts on each array was computed. The differential expression was assessed using linear models and empirical Bayes statistics implemented in the limma package/R (Ritchie et al. 2015). To account for multiple testing, the *p*‐value was adjusted using the Benjamini‐Hochberg method. The genes with a minimum 1.5‐fold change in expression between groups and an adjusted *p*‐value less than 0.05 were considered differentially expressed. The differentially expressed genes (DEGs) were visualized using a volcano plot and then used to build a supervised hierarchical cluster with a heatmap in SRplot software (Tang et al. 2023). All microarray data used in this study are publicly available in the Gene Expression Omnibus (GEO) repository under accession number GSE 307516.

### 2.6 Functional Analysis of Differentially Expressed Genes (DEGs)

The dataset containing differentially expressed genes was uploaded into ingenuity pathway analysis software (IPA, QIAGEN Inc.), and queried against a reference set specific to Agilent Whole Human Genome 4 × 44 k arrays IPA Core Analysis function was used to identify key molecular and cellular functions, and canonical pathways relevant to our dataset, and to predict the main upstream regulators that may influence the transcription or expression of the DEGs. The significance of the associations between the input genes and functions or pathways was estimated by the Fisher Exact test (*p* < 0.05) and a cutoff score greater than 1.3. The IPA regulation *z*‐score algorithm was used to predict the activation states of upstream or downstream processes, where a *z*‐score higher than 2 or smaller than −2 is considered significant.

Gene set enrichment analysis (GSEA) was performed using DEGs ranked by signal intensity, with statistical significance assessed through 1000 gene set permutations in the H (hallmark gene sets) collection from MSigDB (Molecular Signatures Database) (Liberzon et al. 2015). This approach was used to gain a deeper understanding of the molecular mechanisms and functions in the DEGs dataset. Statistical significance was defined by a *p*‐value of < 0.01 and a false discovery rate (FDR) with a *q*‐value of < 0.05.

Gene Ontology (GO) Enrichment Analysis powered by PANTHER 19.0 online software (https://pantherdb.org) was used to find the molecular functions, cellular components, and biological processes over‐represented in our DEGs dataset (FR cutoff of 1.5 and an adjusted *p*‐value of 0.05) (Thomas et al. 2022).

### 2.7 Microarray Data Validation by Real‐Time Quantitative Reverse Transcription PCR (qRT‐PCR)

500 ng total RNA was used for cDNA synthesis using the First Strand cDNA synthesis kit (Hoffmann‐La Roche, Basel, Switzerland) following the random hexamer primer protocol. The cDNAs were diluted 1:10 (v/v) and amplified with TaqMan Master kit (Hoffmann‐La Roche) in a final volume of 20 μL using a LightCycler 480 II thermocycler (Hoffmann‐La Roche). According to the manufacturer's recommendations, the PCR amplification was performed with 1 μM specific primers (Tib Molbiol) and a 0.2 μM specific hydrolysis probe from the Universal Probe Library (UPL). The relative expression levels of target genes (LSBE vs. CTR) were quantified by the ΔΔCt method using 18S as the housekeeping gene.

### 2.8 Statistical Analysis

Student's *t*‐test was used to determine the statistically significant differences between gene expressions modulated by LSBE treatment and validated by qRT‐PCR analysis. *p*‐values less than 0.05 were considered significant. Venn diagrams were done in Venny 2.1.0 software (Oliveros 2007–2015) Venny. An interactive tool for comparing lists with Venn's diagrams (https://bioinfogp.cnb.csic.es/tools/venny/index.html), while the qRT‐PCR graph was illustrated using GraphPad Prism 8 Software (GraphPad Software Inc., Avenida de la Playa La Jolla, San Diego, CA, USA).

## 3 Results

### 3.1 LSBE Treatment Induces Changes in the Gene Expression Profile of BT‐549 Triple‐Negative Breast Cancer Cells

**FIGURE 1:**
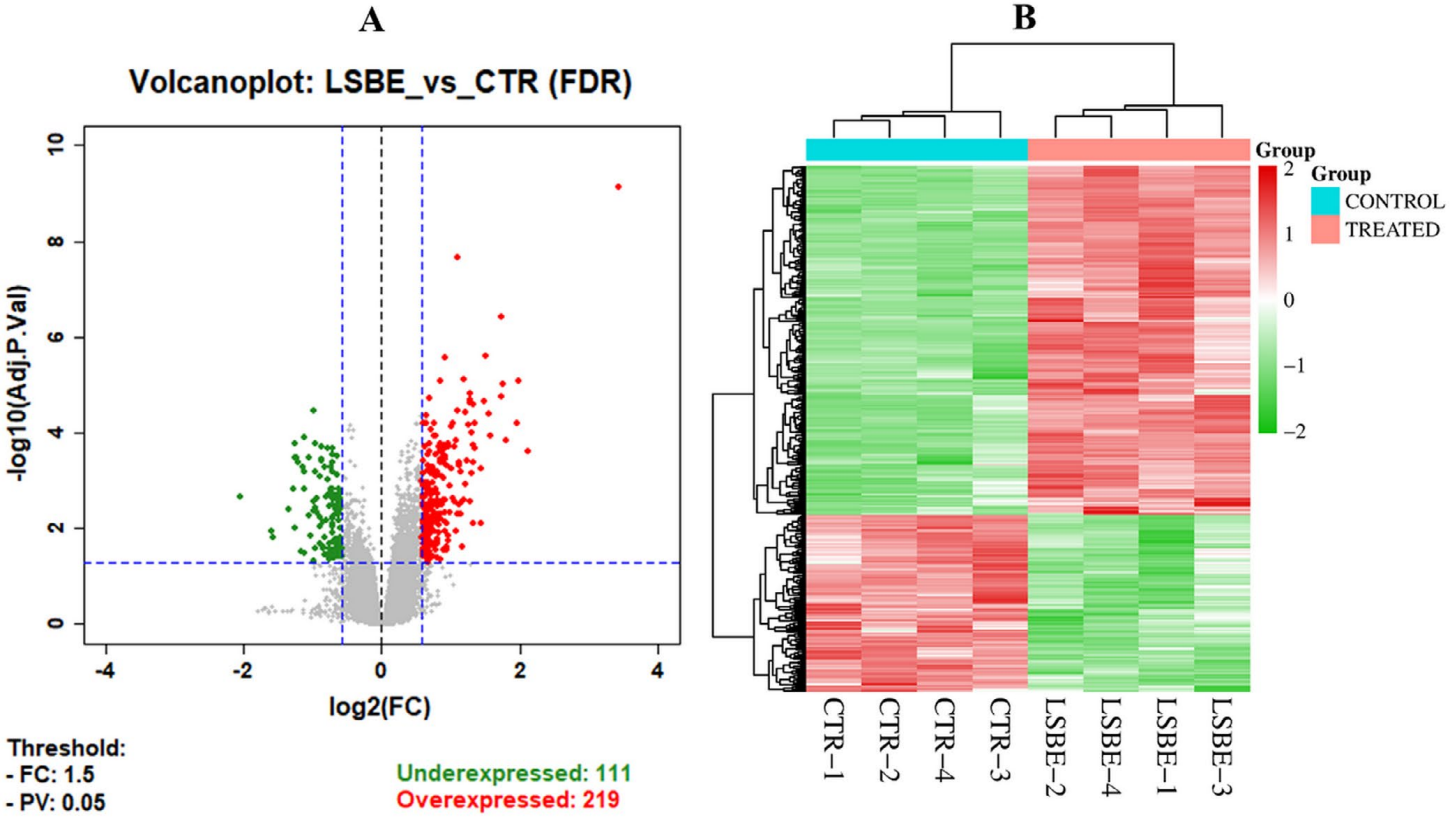
(A) The volcano plot reveals the DEGs identified in BT‐549 cells treated with 4 μM LSBE for 24 h compared to the control group, at an FR cutoff of 1.5 and an adjusted *p*‐value of 0.05. The genes in green are underexpressed (111), and the genes in red are overexpressed (219); (B) Heatmap of 330 DEGs between CTR (*n* = 4) and LSBE (*n* = 4) samples obtained from supervised hierarchical clustering using Euclidean distances. The color indicates the level of mRNA expression: red = higher level of expression, green = lower level of expression, and white expression changes (each row represents a gene, and each column represents a sample). The control (CTR) samples were clustered and separated from the LSBE samples.

Microarray analysis of BT‐549 cells treated with LSBE identified 29,162 annotated genes, of which 330 met the criteria for significant differential expression (fold change > 1.5, adjusted *p* < 0.05) (Table S1, Figure 1A). Among these, 219 were upregulated and 111 downregulated (Table S2). Most changes (79%) showed a moderate amplitude (< 2‐fold), with expression values ranging from −4.14 to 10.84. Hierarchical clustering based on these DEGs revealed a clear separation between LSBE‐treated and control samples, underscoring the transcriptional impact of LSBE (Figure 1B).

### 3.2 Evaluation of Genome‐Wide Expression Profiles Using Ingenuity Pathways Analysis (IPA)

Functional profiling with IPA revealed that LSBE‐induced gene expression changes were strongly linked to cancer‐related processes (*p* = 2.32E−03–1.43E−15), with cellular development (*p* = 2.24E−03–1.45E−14) emerging as the top function. Canonical pathway analysis highlighted cholesterol biosynthesis as the most significantly activated pathway (*p* = 2.69E−15). The top five enriched pathways, diseases, and biological functions regulated by LSBE in BT‐549 cells are summarized in Table 1.

**TABLE 1:** The top 5 significant canonical pathways, diseases, and bio functions, respectively, molecular and cellular functions modulated by LSBE treatment in BT‐549 cells, as identified by IPA.

Diseases and disorders	*p* range	Molecules
Cancer	2.32E−03–1.43E−15	297
Organismal injury and abnormalities	2.32E−03–1.43E−15	306
Dermatological diseases and conditions	1.54E−03–8.69E−10	69
Cardiovascular disease	2.04E−03–2.19E−09	128
Neurological disease	1.94E−03–2.19E−09	156

Molecular and cellular functions	*p* range	Molecules
Cellular development	2.24E−03–1.45E−14	182
Cellular growth and proliferation	2.24E−03–1.45E−14	171
Cell death and survival	2.29E−03–2.98E−12	171
Lipid metabolism	2.24E−03–3.49E−11	98
Small molecule biochemistry	2.24E−03–3.49E−11	122

Canonical pathways	*p*	Overlap
Cholesterol biosynthesis	2.69E−15	48% (12/25)
Activation of gene expression by SREBF (SREBP)	2.29E−12	30% (12/40)
Superpathway of cholesterol biosynthesis	2.22E−11	37% (10/28)
Response of EIF2AK1 (HRI) to heme deficiency	2.60E−09	46.7% (7/15)
Ferroptosis signaling pathway	5.34E−09	11.9% (15/126)

**FIGURE 2:**
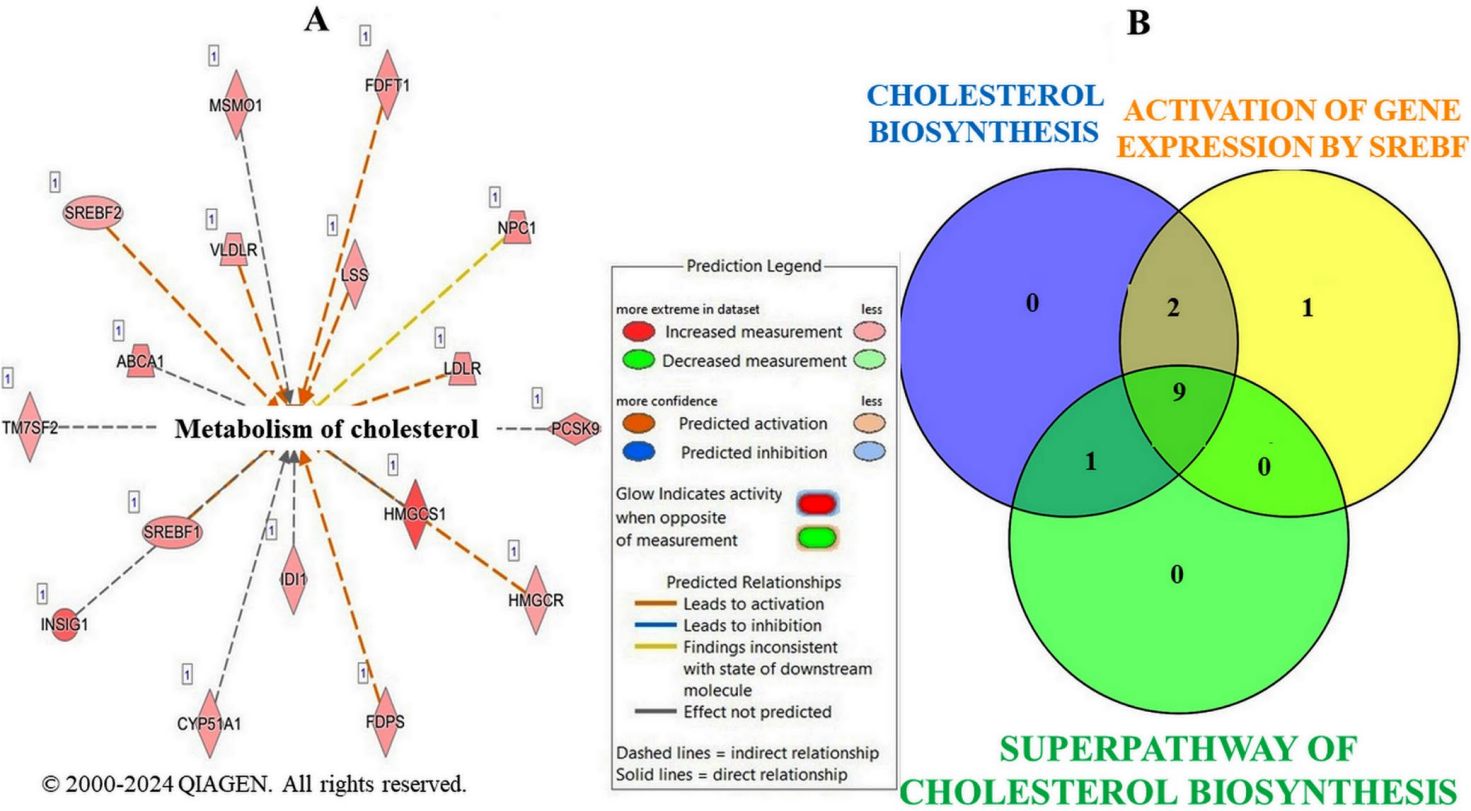
(A) Genes involved in the metabolism of cholesterol identified by IPA; (B) Venn diagram of the overlap of the differently expressed genes involved in cholesterol biosynthesis (*n* = 12), activation of gene expression by SREBF (SREBP) (*n* = 12), and superpathway of cholesterol biosynthesis (*n* = 10) modulated by LSBE treatment in BT‐549 cells identified with IPA.

Upon further IPA analysis of the DEGs modulated by LSBE treatment, we found that cholesterol metabolism (*z*‐score = 0.251, *p*‐value = 5.73E−10) identified by IPA contains 17 key upregulated genes from our data set: ABCA1, CYP51A1, FDFT1, FDPS, HMGCR, HMGCS1, IDI1, INSIG1, LDLR, LSS, MSMO1, NPC1, PCSK9, SREBF1, SREBF2, TM75SF2, and VLDLR (Figure 2A).

Cholesterol biosynthesis emerged as the most significantly activated canonical pathway in IPA (*z*‐score = 3.464, *p* = 2.69E−15, ratio = 12/25), with 12 of 25 key genes upregulated in our dataset (Table 2).

**TABLE 2:** The upregulated genes involved in cholesterol biosynthesis modulated by LSBE treatment in BT‐549 TNBC cells, as identified by IPA.

Cholesterol biosynthesis		Molecules = 12	*z*‐score = 3.464	*p* = 2.69E−15	Ratio = 12/25 (0.48)	
Gene symbol	Gene name	Identifier	Fold regulation	Expected	Location	Type
CYP51A1	Cytochrome P450 family 51 subfamily A member 1	NM_000786	1.733	Up	Cytoplasm	Enzyme
FDFT1	Farnesyl‐diphosphate farnesyltransferase 1	NM_004462	1.893	Up	Cytoplasm	Enzyme
FDPS	Farnesyl diphosphate synthase	NM_002004	1.799	Up	Cytoplasm	Enzyme
HMGCR	3‐Hydroxy‐3‐methylglutaryl‐CoA reductase	NM_000859	1.584	Up	Cytoplasm	Enzyme
HMGCS1	3‐Hydroxy‐3‐methylglutaryl‐CoA synthase 1	NM_002130	3.346	Up	Cytoplasm	Enzyme
IDI1	Sopentenyl‐diphosphate delta isomerase 1	NM_004508	1.689	Up	Cytoplasm	Enzyme
LSS	Lanosterol synthase	NM_002340	1.677	Up	Cytoplasm	Enzyme
MSMO1	Methylsterol monooxygenase 1	NM_006745	1.795	Up	Cytoplasm	Enzyme
MVD	Mevalonate diphosphate decarboxylase	NM_002461	1.599	Up	Cytoplasm	Enzyme
SREBF1	Sterol regulatory element‐binding transcription factor 1	NM_001005291	1.948	Up	Nucleus	Transcription regulator
SREBF2	Sterol regulatory element‐binding transcription factor 2	NM_004599	1.516	Up	Nucleus	Transcription regulator
TM7SF2	Transmembrane 7 superfamily member 2	NM_003273	1.559	Up	Cytoplasm	Enzyme

We also found significant activation of two important pathways that are regulated by cholesterol biosynthesis as follows: Activation of gene expression by SREBF (SREBP) (12 molecules, *z*‐score = 3.464, *p*‐value = 2.29E−12) and Superpathway of Cholesterol Biosynthesis (10 molecules, *z*‐score = 3.161, *p*‐value = 2.22E−11).

Overlap analysis revealed shared regulation of cholesterol biosynthesis, SREBF‐mediated gene expression, and the Superpathway of Cholesterol Biosynthesis, with nine core genes (e.g., HMGCR, FDFT1, LSS) consistently upregulated (Figure 2B). LSBE treatment also activated ferroptosis, a lipid peroxidation–driven form of cell death (Yan et al. 2021). IPA analysis (*z*‐score = 0.775, *p* = 5.34E−09) revealed 15 upregulated genes encoding enzymes, transporters, and transcription regulators, mainly localized to the plasma membrane and cytoplasm (Table 3; Figure S1).

**TABLE 3:** The upregulated genes involved in ferroptosis, modulated by LSBE treatment in BT‐549 TNBC cells, as identified by IPA.

Ferroptosis		molecules = 15	*z*‐score = 0.775	*p* = 5.34E−09	ratio = 15/126 (0.119)	
Gene symbol	Gene name	Identifier	Fold regulation	Expected	Location	Type
ABCA1	ATP‐binding cassette subfamily A member 1	NM_005502	2.148	Up	Plasma membrane	Transporter
ANGPTL4	Angiopoietin like 4	NM_139314	2.714	Up	Extracellular space	Other
ATF4	Activating transcription factor 4	NM_001675	1.732	Up	Nucleus	Transcription regulator
CBS/LOC102724560	Cystathionine‐beta‐synthase like	NM_000071	1.584	Down	Cytoplasm	Enzyme
CHAC1	ChaC glutathione specific gamma‐glutamylcyclotransferase 1	NM_024111	2.494	Up	Cytoplasm	Enzyme
EMP1	Epithelial membrane protein 1	NM_001423	1.679	Up	Plasma membrane	Other
FDFT1	Farnesyl‐diphosphate farnesyltransferase 1	NM_004462	1.893	Up	Cytoplasm	Enzyme
FTL	Ferritin light chain	NM_000146	1.527	Up	Cytoplasm	Enzyme
HMGCR	3‐Hydroxy‐3‐methylglutaryl‐coa reductase	NM_000859	1.584	Up	Cytoplasm	Enzyme
HMOX1	Heme oxygenase 1	NM_002133	1.897	Up	Cytoplasm	Enzyme
SAT1	Spermidine/Spermine N1‐acetyltransferase 1	NM_002970	1.896	Up	Cytoplasm	Enzyme
SLC3A2	Solute carrier family 3 member 2	NM_001012662	1.825	Down	Plasma membrane	Transporter
SLC7A11	Solute carrier family 7 member 11	NM_014331	1.577	Down	Plasma membrane	Transporter
SQSTM1	Sequestosome 1	NM_003900	1.588	Down	Cytoplasm	Transcription regulator
SREBF2	Sterol regulatory element‐binding transcription factor 2	NM_004599	1.516	Down	Nucleus	Transcription regulator

**FIGURE 3:**
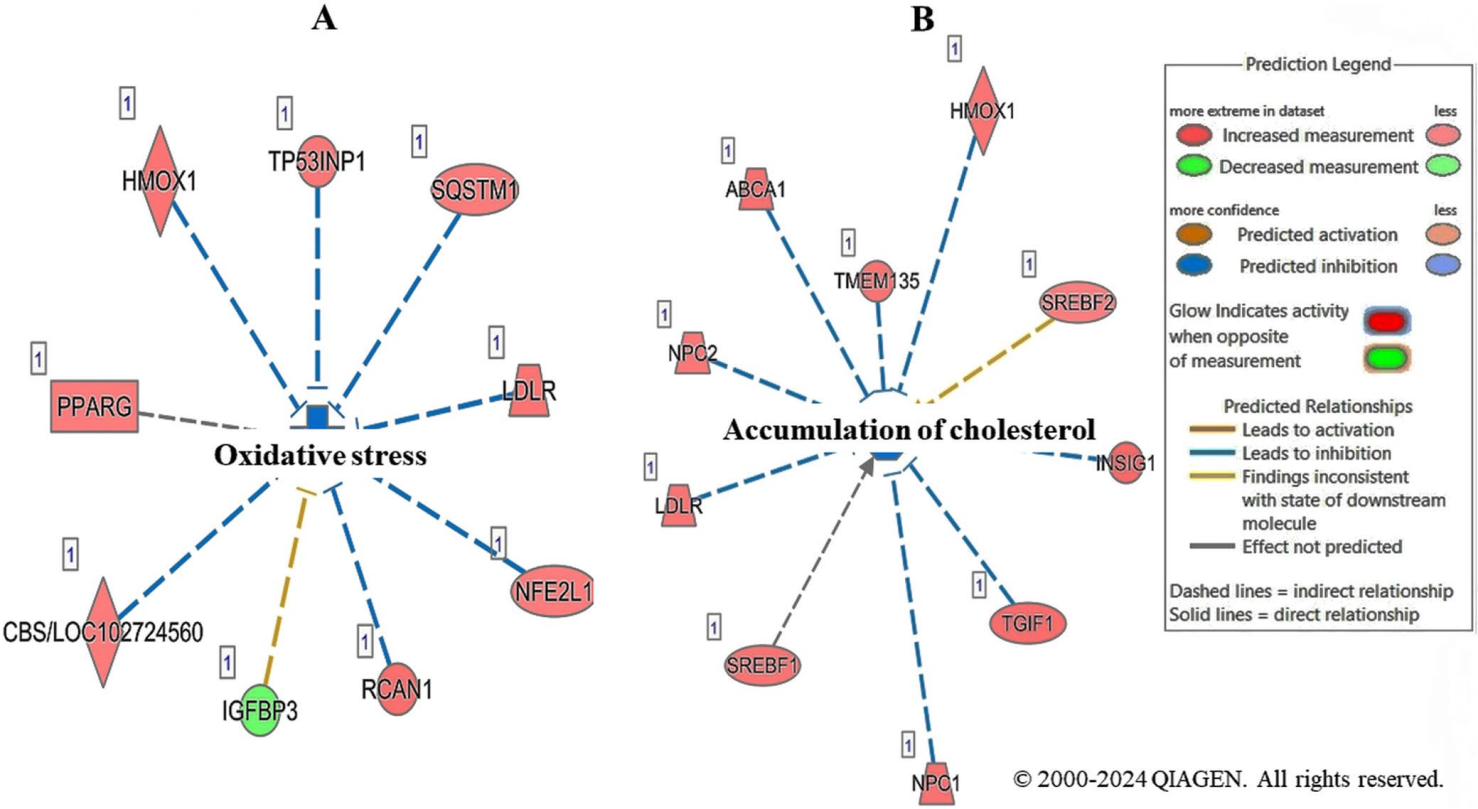
Molecular networks of (A) oxidative stress and (B) accumulation of cholesterol identified with ingenuity pathway analysis (IPA) and modulated LSBE in BT‐549 cells based on the gene expression profile.

Consistent with our previous findings that LSBE (IC_50_) induced early apoptosis (15.4%), late apoptosis (40.6%), and necrosis (8.3%) in BT‐549 cells, IPA analysis identified 51 genes linked to apoptosis and necrosis (Table S3). LSBE treatment in BT‐549 cells significantly inhibited oxidative stress (*z*‐score = −2.180, *p* = 3.38E−05) and cholesterol accumulation (*z*‐score = −2.140, *p* = 3.36E−06) (Tables 4 and 5, Figure 3).

**TABLE 4:** The DEGs involved in oxidative stress, modulated by LSBE treatment in BT‐549 TNBC cells, as identified by IPA.

Oxidative stress		*z*‐score = −2.180		*p* = 3.83E−05		
Gene symbol	Gene name	Identifier	Fold regulation	Prediction	Location	Type
CBS/LOC10272	Cystathionine‐beta‐synthase like	NM_000071	1.584	Decreased	Cytoplasm	Enzyme
HMOX1	Heme oxygenase 1	NM_002133	1.897	Decreased	Cytoplasm	Enzyme
IGFBP3	Insulin like growth factor‐binding protein 3	NM_001013398	−1.981	Increased	Extracellular space	Other
LDLR	Low density lipoprotein receptor	NM_000527	1.895	Decreased	Plasma membrane	Transporter
NFE2L1	NFE2 like BZIP transcription factor 1	NM_003204	1.61	Decreased	Nucleus	Transcription regulator
PPARG	Peroxisome proliferator activated receptor gamma	NM_015869	1.581	Affected	Nucleus	Ligand‐dependent nuclear receptor
RCAN1	Regulator of calcineurin 1	NM_004414	2.331	Decreased	Nucleus	Other
SQSTM1	Sequestosome 1	NM_003900	1.588	Decreased	Cytoplasm	Transcription regulator
TP53INP1	Tumor protein P53 inducible nuclear protein 1	NM_033285	1.766	Decreased	Nucleus	Other

**TABLE 5:** The DEGs involved in the accumulation of cholesterol, modulated by LSBE treatment in BT‐549 TNBC cells, as identified by IPA.

Accumulation of cholesterol			*z*‐score = −2.140		*p* = 3.36E−06	
Gene symbol	Gene name	Identifier	Fold regulation	Prediction	Location	Type
ABCA1	ATP‐binding cassette subfamily A member 1	NM_005502	2.148	Decreased	Plasma membrane	Transporter
HMOX1	Heme oxygenase 1	NM_002133	1.897	Decreased	Cytoplasm	Enzyme
INSIG1	Insulin‐induced gene 1	NM_198336	2.939	Decreased	Cytoplasm	Other
LDLR	Low density lipoprotein receptor	NM_000527	1.895	Decreased	Plasma membrane	Transporter
NPC1	NPC intracellular cholesterol transporter 1	NM_000271	2.067	Decreased	Cytoplasm	Transporter
NPC2	NPC intracellular cholesterol transporter 2	NM_006432	1.754	Decreased	Extracellular space	Transporter
SREBF1	Sterol regulatory element‐binding transracellular cholesterol transporter 1	NM_000271	2.067	Decreased	Cytoplasm	Transporter
NPC2	NPC intracellular cholesterol transporter 2	NM_006432	1.754	Decreased	Extracellular space	Transporter
SREBF1	Sterol regulatory element‐binding transcription factor 1	NM_001005291	1.948	Affected	Nucleus	Transcription regulator
SREBF2	Sterol regulatory element‐binding transcription factor 2	NM_004599	1.516	Increased	Nucleus	Transcription regulator
TMEM135	Transmembrane protein 135	NM_022918	2.435	Decreased	Nucleus	Transcription regulator

### 3.3 Upstream Analysis of DEGs Following LSBE Treatment

**FIGURE 4:**
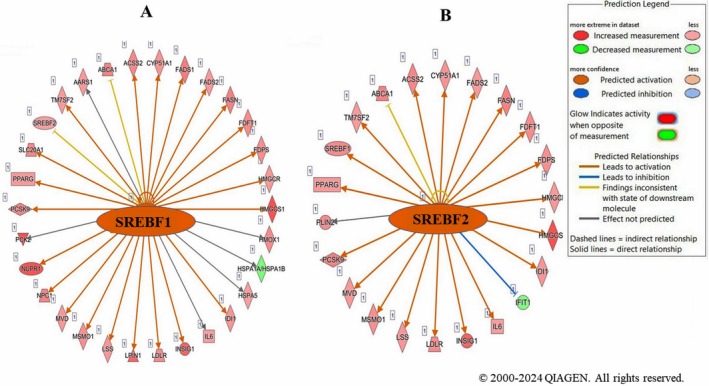
Molecular networks of the upstream regulators (A) SREBF1 and (B) SREBF2 predicted by ingenuity pathway analysis (IPA) and modulated by LSBE in BT‐549 cells based on the gene expression profile.

IPA upstream regulator analysis identified SREBF1 (FR = 1.94, *z* = 4.326, *p* = 9.97E−17) and SREBF2 (FR = 1.51, *z* = 3.885, *p* = 9.97E−19) as significantly activated, driving target gene expression (Figure 4; Table S4).

**FIGURE 5:**
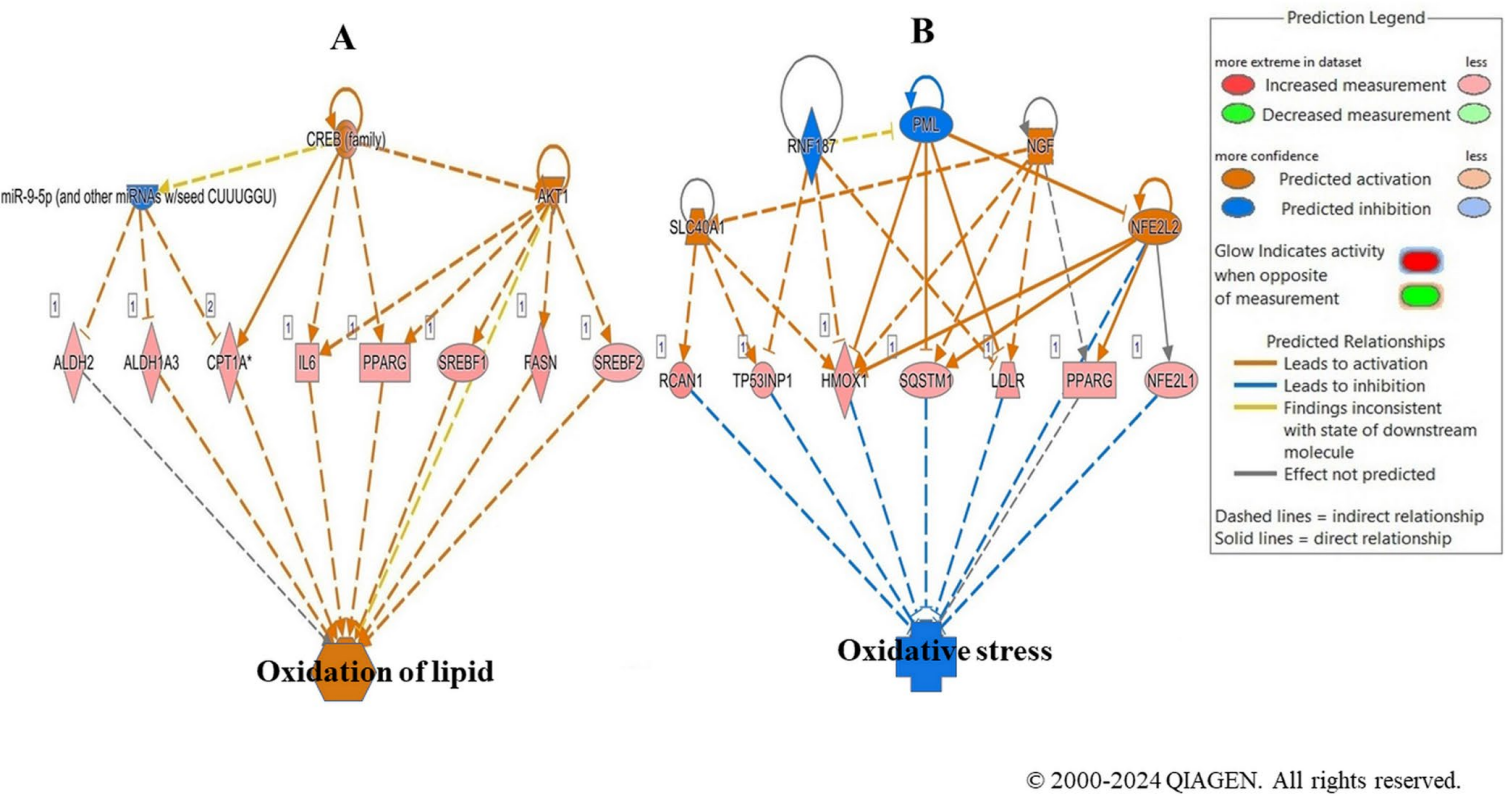
Molecular networks of the downstream regulators involved in (A) oxidation of lipids and (B) oxidative stress predicted by ingenuity pathway analysis (IPA), and modulated by LSBE in BT‐549 cells based on the gene expression profile.

IPA effector analysis predicted that CREB family and AKT1 drive lipid oxidation via upregulation of ALDH2, CPT1A, SREBF1/2, and FASN, while SLC40A1, NGF, and NFE2L2 activation, together with RNF187 and PML inhibition, promote oxidative stress suppression through their target genes, including HMOX1, SQSTM1, and PPARG (Figure 5).

### 3.4 Evaluation of Genome‐Wide Expression Profiles Using Gene Set Enrichment Analysis (GSEA)

**FIGURE 6:**
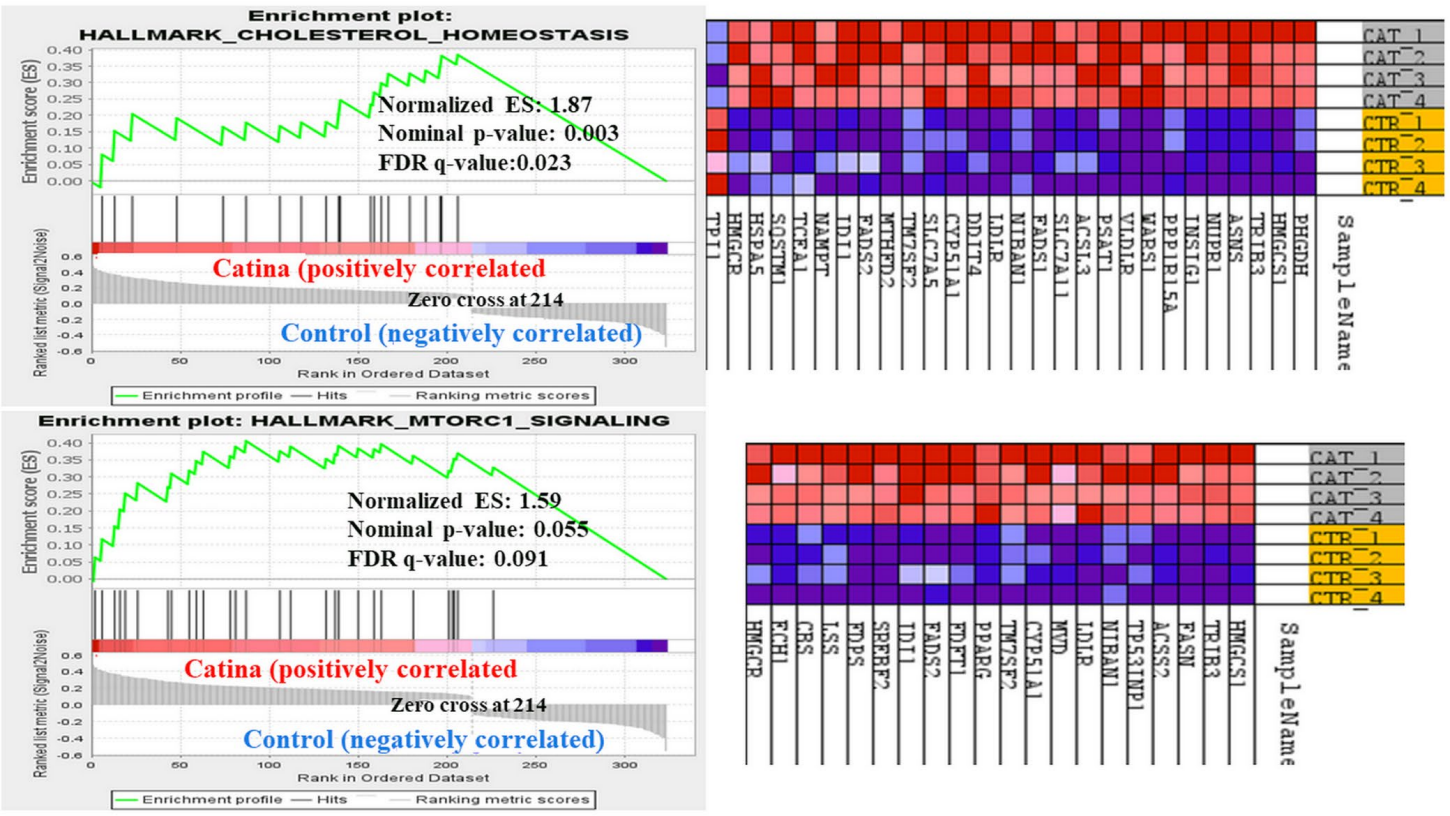
The GSEA analysis of cells treated with 4 μM LSBE for 24 h. All the signaling pathways identified in the H: hallmark gene sets are enriched and upregulated.

GSEA of DEGs revealed that LSBE (IC_50_) treatment upregulated hallmark gene sets linked to cholesterol homeostasis and mTORC1 signaling in BT‐549 cells. (Figure 6).

**FIGURE 7:**
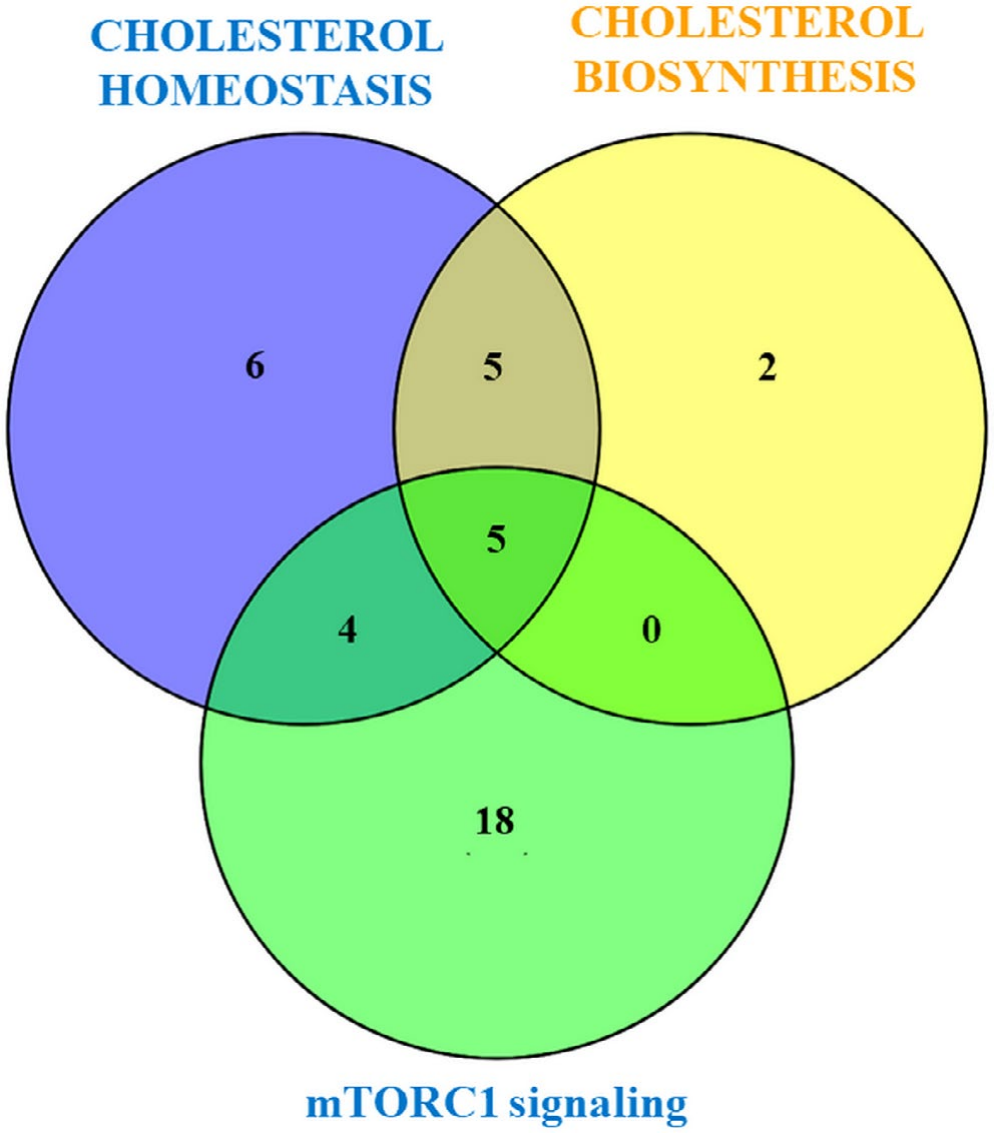
Venn diagram of the overlap of the differently expressed genes involved in cholesterol homeostasis (*n* = 20), mTORC1 signaling (*n* = 27), identified with GSEA, respectively cholesterol biosynthesis (*n* = 12), identified with IPA software, and modulated by LSBE treatment in BT‐549 cells.

Cholesterol has been shown to regulate mTORC1 signaling via the lysosomal protein LYCHOS (Shin et al. 2022). By overlapping IPA and GSEA results, we identified shared genes linking cholesterol biosynthesis, homeostasis, and mTORC1 signaling, including HMGCR, HMGCS1, and SREBF2 (Figure 7).

### 3.5 Evaluation of Genome‐Wide Expression Profiles Using Gene Ontology (GO) Enrichment Analysis

GO enrichment analysis of LSBE‐treated BT‐549 cells highlighted biological processes such as cellular process (24.5%), biological regulation (17%), and metabolic process (9.5%). Most DEGs were linked to binding (27.1%) and catalytic activity (17.4%). GO Pathway analysis revealed enrichment in apoptosis (2.8%), TGF‐β, Wnt signaling (2.1%), and cholesterol biosynthesis (1.4%), while GO Reactome‐based analysis identified significant enrichment in metabolism (31 genes, FDR = 9.73E−03) and cellular response to stimuli (16 genes, FDR = 2.97E−02) (Table S5).

### 3.6 qRT‐PCR Validation of the Microarray Data

**FIGURE 8:**
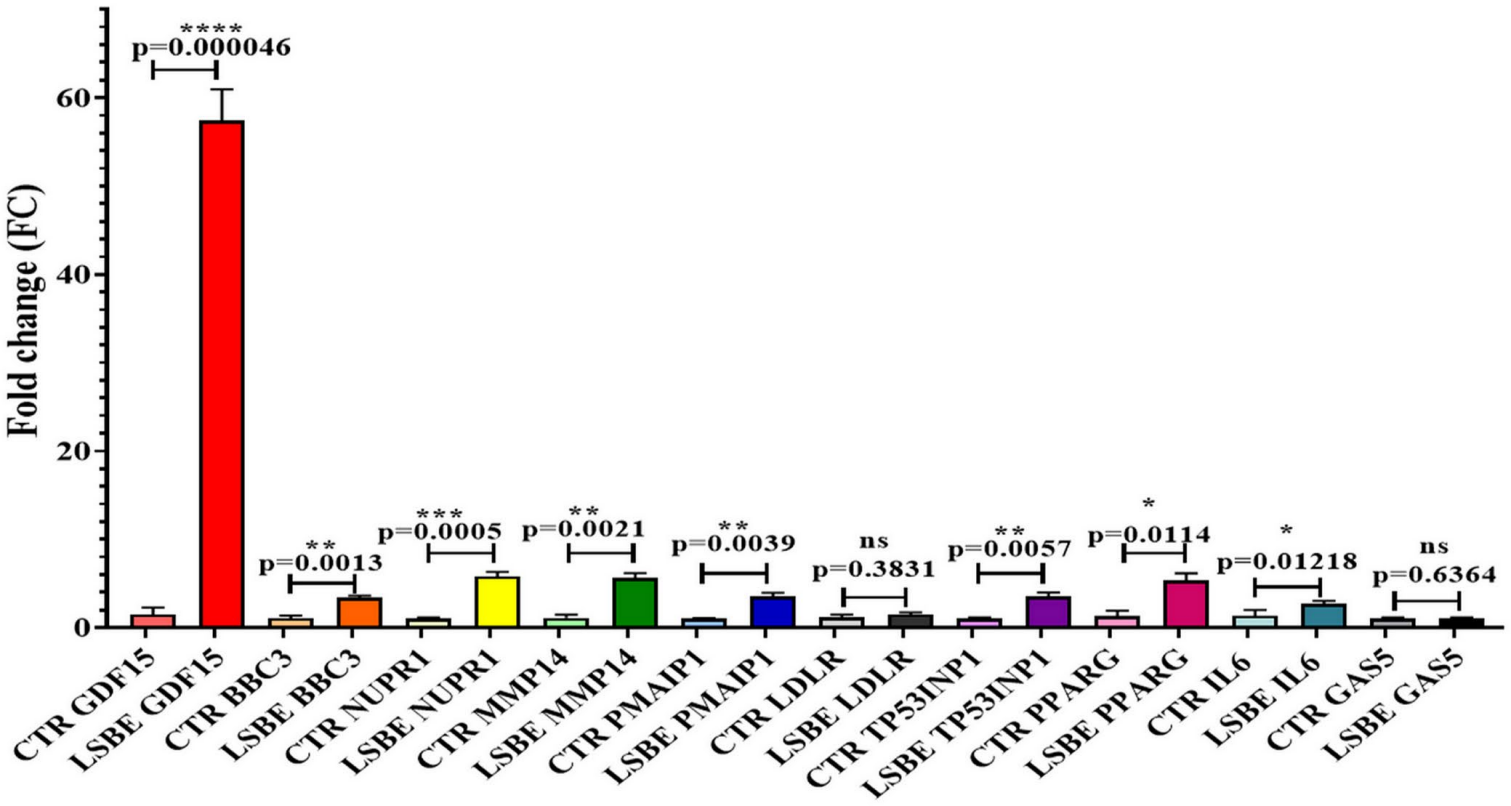
qRT‐PCR validation of the microarray data containing differentially expressed genes from oxidative stress, necrosis, and apoptosis. Values are determined as means ± SEM from four biological replicates. *p < 0.05, **p < 0.01, ***p < 0.001, ****p < 0.0001 compared to the control.

qRT‐PCR validation of 10 genes related to apoptosis, necrosis, oxidative stress, cholesterol homeostasis, and mTORC1 signaling confirmed microarray results, with all showing significance except GAS5 (FR = 1.06) and LDLR (FR = 1.31) (Figure 8, Table 6).

**TABLE 6:** Overlap between the microarray and qRT‐PCR results.

Gene	GDF15	BBC3	NUPR1	MMP14	PMAIP1	LDLR	TP53INP1	PPARG	IL6	GAS5
FR qRT‐PCR	38.36	3.13	5.71	5.04	3.50	1.32	3.47	4.04	1.96	1.06
*p* qRT‐PCR	4.7E−05	1.3E−03	5.1E−04	2.1E−03	4.0E−03	3.8E−01	5.7E−03	1.1E−02	1.2E−01	6.4E−01
FR microarray	10.84	3.49	3.35	2.50	1.89	1.89	1.77	1.58	1.53	0.64
*p* microarray	2E−14	3E−07	7E−09	1E−04	2E−06	4E−07	2E−04	8E−07	4E−04	2E+00

## 4 Discussion

Several in vitro studies report that sea buckthorn extracts from seeds, berries, leaves, or juice inhibit breast cancer cell proliferation and induce apoptosis via mechanisms such as CASP3, IGFBP4, GADD34 modulation, cell cycle arrest, and TNF–NF‐κB signaling inhibition (Olas et al. 2018), (Xu et al. 2024). While most work has focused on phenolic compounds, seed procyanidins trigger apoptosis in MDA‐MB‐231 cells through fatty acid synthase inhibition (Wang et al. 2014), and flavonoids like isorhamnetin, quercetin, and kaempferol suppress oncogenic PI3K‐Akt–mTOR and NF‐κB signaling (Martiniakova et al. 2024). Although carotenoids are key constituents of sea buckthorn, no studies have examined the carotenoid fraction specifically in TNBC cells. Evidence from other sources shows carotenoids exert anticancer effects, often with selectivity for TNBC: for instance, β‐carotene strongly inhibited MDA‐MB‐231 growth and migration via JNK‐mediated S‐phase arrest and apoptosis, while sparing MCF‐12A normal breast epithelial cells and enhancing doxorubicin efficacy (Antunes et al. 2022). Recent studies show that carotenoids exert selective anticancer effects against TNBC cells: β‐carotene and related pigments from *Rhodosporidium* sp. induced apoptosis and cell cycle arrest in MDA‐MB‐231 cells with low toxicity to normal cells (Sinha et al. 2023); carotenoids isolated from *Paracoccus* sp., containing 48.3% zeaxanthin (the major compound of LSBE), promoted apoptosis via BAX/BCL‐2 modulation and showed strong affinity (Abdelazim et al. 2025); and fucoxanthin suppressed proliferation, migration, and VEGF signaling in MDA‐231 TNBC cells (Ahmed et al. 2023). Consistent with our previous findings, LSBE (IC₅₀ = 12.62 μM) induced multiple forms of programmed cell death in BT‐549 cells, including early apoptosis (15.4%), late apoptosis (40.6%), and necrosis (8.3%), highlighting its ability to trigger diverse cytotoxic mechanisms in TNBC. However, no study to date has examined the transcriptional profiles induced by carotenoid treatment in breast cancer, despite systematic reviews outlining their antitumor mechanisms (Giani et al. 2021; Baeza‐Morales et al. 2024).

Cholesterol is essential for cell structure, hormone synthesis, and homeostasis, with its role in breast cancer remaining controversial, where some studies link high levels to increased risk, while others show no or even inverse associations, particularly with HDL (Ben Hassen et al. 2023). Cholesterol metabolism involves biosynthesis, uptake, efflux, and esterification, tightly regulated by SREBP‐2 through genes such as HMGCR, LDLR, ABCA1, and ABCG1 (Xiao et al. 2023). LSBE treatment in BT‐549 TNBC cells upregulated key genes in cholesterol biosynthesis and homeostasis, including HMGCR, SREBF2, LDLR, and ABCA1, suggesting enhanced cholesterol regulation with reduced oxidative stress and cholesterol accumulation. Our findings suggest that LSBE‐induced activation of SREBF‐driven gene expression and the cholesterol biosynthesis pathway may expose a metabolic susceptibility in TNBC, consistent with emerging evidence that targeting metabolic reprogramming could offer therapeutic opportunities in aggressive breast cancer subtypes (X. Cai et al. 2025). Nevertheless, we observed in our transcriptomic study the apparent discrepancy between the upregulation of cholesterol biosynthesis and the inhibition of cholesterol accumulation. This dual effect may reflect a compensatory mechanism, whereby SREBF1/2‐driven transcription enhances biosynthetic gene expression while simultaneously upregulating cholesterol efflux and uptake regulators such as ABCA1 and LDLR prevent intracellular cholesterol overload, thereby maintaining cellular homeostasis.

The 3‐hydroxy‐3‐methyl glutaryl coenzyme A reductase (HMGCR) is the primary rate‐limiting enzyme in cholesterol biosynthesis that catalyzes HMG‐CoA reduction to MVA. A recent study found no association between cancer mortality and HMGCR expression or statin use (Kuldeep et al. 2023). Our findings suggest that LSBE‐driven upregulation of HMGCR, a statin‐targeted enzyme, under SREBF1/2 control, thus highlighting a potential therapeutic axis linking cholesterol metabolism with ferroptosis and mTORC1 signaling in TNBC. Therefore, by modulating SREBF1/2–HMGCR signaling, LSBE may expose a targetable metabolic susceptibility in TNBC, offering opportunities to integrate metabolic interventions with existing therapies (Hillis et al. 2024).

mTORC1 regulates metabolic homeostasis through anabolic–catabolic balance to prevent disease and extend health span (Szwed et al. 2021). Our data suggests that LSBE‐induced activation of SREBF1/2 drives transcriptional upregulation of key genes in cholesterol metabolism that also intersect with mTORC1 signaling. We hypothesize that by linking SREBF1/2‐driven cholesterol metabolism with mTORC1 signaling, LSBE treatment highlights a potential metabolic vulnerability in TNBC that could be therapeutically investigated (Yang et al. 2025).

Ferroptosis, a non‐apoptotic form of cell death driven by lipid peroxidation and ROS accumulation, has emerged as a key susceptibility in cancer cells (Forcina and Dixon 2019). In our transcriptional study, LSBE treatment upregulated a set of lipid oxidation genes (ALDH2, ALDH1A3, CPT1A, IL6, PPARG, SREBF1, FASN, SREBF2), with SREBF2 identified as a central upstream regulator linking ferroptosis and lipid metabolism. Overlapping gene sets further confirmed the involvement of ABCA1, FDFT1, HMGCR, and IL6, suggesting that LSBE promotes ferroptosis via SREBF2‐driven lipid oxidation. These findings align with prior evidence showing ROS‐mediated lipid peroxidation reduces cancer cell proliferation and viability (Conrad and Pratt 2019). Importantly, since ferroptosis‐enhancing agents have been proposed to overcome cisplatin resistance in TNBC (Lee et al. 2021), (Mirzaei et al. 2021) and phytochemicals such as curcumin, emodin, and chloroquine have demonstrated similar chemosensitizing effects (Hong et al. 2020), emodin (Ding et al. 2018), chloroquine (Qu et al. 2017). Our findings suggest that carotenoid‐rich LSBE may act as a ferroptosis‐inducing chemosensitizer, enhancing the efficacy of standard TNBC therapies such as cisplatin. This notion is supported by recent work showing that TNBCs with high expression of fatty acid desaturases FADS1/2 are particularly susceptible to ferroptosis‐inducing agents, both in vitro and in vivo (Lorito et al. 2024). Together, these data point toward a metabolic susceptibility in TNBC that could be exploited therapeutically by combining LSBE (or similar carotenoid‐rich extracts) with ferroptosis inducers or chemotherapy.

Oxidative stress arises from an imbalance between ROS production and antioxidant defenses, driving DNA damage, genomic instability, and cancer progression (Sies et al. 2022), (Rizvi et al. 2021). Nonenzymatic antioxidants such as β‐carotene, vitamins C and E, and glutathione play a key role in maintaining cellular homeostasis (Gyurászová et al. 2020; Birben et al. 2012; di Martino et al. 2018). A recently published study performed on sea buckthorn‐extracted polyphenols showed a decrease of 88% in ROS generation in TNBC MDA‐231 cells treated with a concentration of 200 μg/mL (Farheen et al. 2024), while our previous study has demonstrated that LSBE, rich in zeaxanthin (42.62%), inhibited ROS production by 51.95% at IC_50_ concentrations in BT‐549 TNBC cells (*p* = 0.0188), reflecting a promising antioxidant capacity. In the present exploratory study, transcriptomic analysis revealed that LSBE carotenoids modulated key nonenzymatic antioxidant pathways by upregulating RCAN1, TP53INP1, HMOX1, SQSTM1, LDLR, PPARG, and NFE2L1, thereby attenuating oxidative stress in TNBC cells, consistent with recent evidence highlighting the antioxidant and cytoprotective role of natural carotenoid extracts (Bufka et al. 2024), emphasizing their potential as complementary agents to improve redox balance in breast cancer therapy.

## 5 Limitations and Future Directions

Our transcriptomic findings suggest that LSBE may engage pathways regulating cholesterol metabolism, oxidative stress, apoptosis, and ferroptosis in TNBC; however, functional validation is critical. Future studies will incorporate ferroptosis‐specific assays (lipid ROS, GSH, MDA, GPX4, and iron‐dependence) and genetic validation of GPX4 to confirm whether LSBE truly induces ferroptotic cell death. Such validation would provide a mechanistic basis for exploring LSBE carotenoids as chemosensitizers or complementary agents to enhance therapeutic efficacy in TNBC. Another key limitation of our study is that the mechanistic conclusions are derived from transcriptomic signatures without direct functional validation. However, the observed link between SREBF1/2‐driven cholesterol metabolism and mTORC1 signaling suggests a potential metabolic susceptibility in TNBC. Given the established role of metabolic reprogramming in therapy resistance, future studies should investigate whether LSBE‐induced modulation of these pathways can be functionally validated and therapeutically exploited, either as a singular therapy or in combination with existing metabolic and chemotherapeutic agents.

## 6 Conclusions

Carotenoid‐rich lipophilic sea buckthorn extract (LSBE) modulated the expression of genes associated with cholesterol biosynthesis, cholesterol homeostasis, mTORC1 signaling, oxidative stress responses, and ferroptosis in BT‐549 TNBC cells. While these transcriptomic signatures suggest potential links to key metabolic and cell death pathways, functional validation will be required to confirm whether these transcriptional changes translate into altered cellular processes. Importantly, the observed upregulation of cholesterol‐ and ferroptosis‐related regulators highlights candidate molecular nodes that may underlie the reported antioxidant and cytotoxic properties of carotenoids. Taken together, our data provide mechanistic hypotheses and molecular targets for future studies investigating LSBE as a potential complementary strategy in triple‐negative breast cancer therapy.

## Author Contributions

**Simona Visan:** conceptualization (equal), visualization (equal), methodology (equal), software (equal), project administration (equal), investigation (equal), writing – original draft (equal), writing – review and editing (equal). **Loredana Balacescu:** methodology (equal), software (equal), data curation (equal), writing – original draft (equal). **Flaviu Drigla:** methodology (equal), writing – original draft (equal). **Ovidiu Balacescu:** methodology (equal), writing – original draft (equal). **Adela Pintea:** supervision (equal), writing – review and editing (equal).

## Ethics Statement

The authors have nothing to report.

## Conflicts of Interest

The authors declare no conflicts of interest.

## Supporting information


**Figure S1:** fsn371112‐sup‐0001‐FigureS1.docx.

**Tables S1–S5:** fsn371112‐sup‐0002‐Tables.xlsx.

## Data Availability

All microarray data used in this study are publicly available in the Gene Expression Omnibus (GEO) repository under accession number GSE 307516, and the gene sets used in IPA and GSEA analyses can be found in Tables S1–S5: DEGs.
