# Supplementary material for: Gene Expression Profiles Modulated by Lipophilic Sea Buckthorn ( Hippophae rhamnoides L.) Extract in BT‐549 Triple‐Negative Breast Cancer Cells
Source: Food Sci Nutr. 2025 Dec 16;13(12):e71112. doi: 10.1002/fsn3.71112 (PMC12706652; doi:10.1002/fsn3.71112)
Supplement: Supplementary file 1 — Figure S1: fsn371112‐sup‐0001‐FigureS1.docx. [file FSN3-13-e71112-s001.docx]

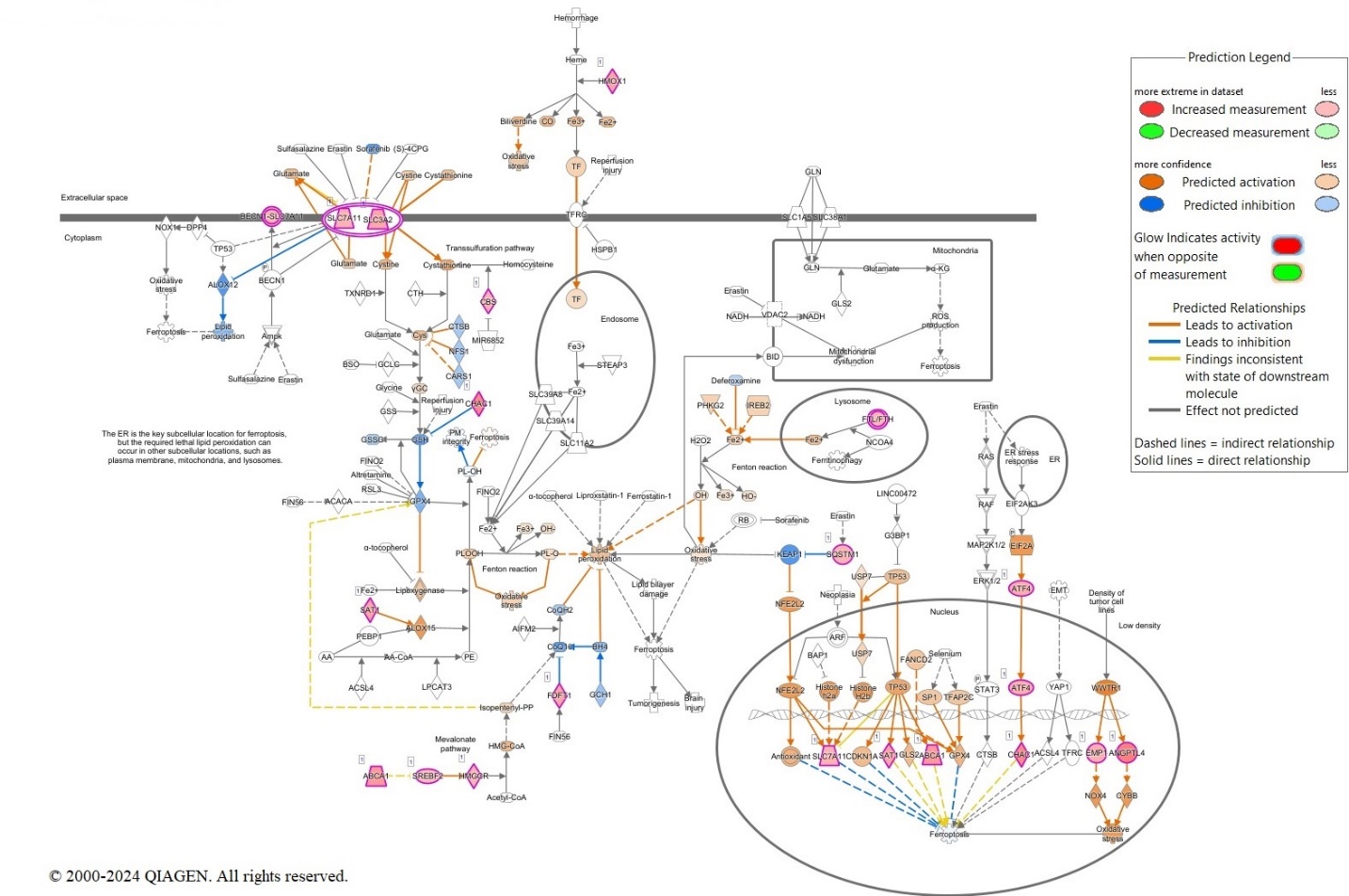


**Figure S1.** Ferroptosis signaling pathway predicted by IPA software as a response of BT-549 cells to LSBE treatment
